# The Gas-Sensing Properties of Ag-/Au-Modified Ti_3_C_2_T_x_ (T=O, F, OH) Monolayers for HCHO and C_6_H_6_ Gases

**DOI:** 10.3390/molecules30020219

**Published:** 2025-01-07

**Authors:** Xinghua Qi, Bahadar Nawab Khattak, Arif Alam, Wenfu Liu, Yingang Gui

**Affiliations:** 1 College of Economics and Management, Huanghuai University, Zhumadian 463000, China; qixinghua@huanghuai.edu.cn; 2 Department of Development Studies, COMSATS University Islamabad, Abbottabad Campus, Abbottabad 22010, Pakistan; arifalam@cuiatd.edu.pk; 3 College of Energy Engineering, Huanghuai University, Zhumadian 463000, China; 4 College of Engineering and Technology, Southwest University, Chongqing 400715, China; yinganggui@swu.edu.cn

**Keywords:** MXene, Ag/Au modification, adsorption, gas-sensing properties, DFT

## Abstract

Based on density functional theory calculations, this study analyzed the gas-sensing performance of Ti_3_C_2_T_x_ (T=O, F, OH) monolayers modified with precious metal atoms (Ag and Au) for HCHO and C_6_H_6_ gas molecules. Firstly, stable structures of Ag- and Au-single-atom doped Ti_3_C_2_T_x_ (T=O, F, OH) surfaces were constructed and then HCHO and C_6_H_6_ gas molecules were set to approach the modified structures at different initial positions. The most stable adsorption structure was selected for further analysis of the adsorption energy, adsorption distance, charge transfer, charge deformation density, total density of states, and partial density of states. The results show that the Ag and Au modifications improved the adsorption performance of Ti_3_C_2_O_2_ for HCHO and C_6_H_6_. In comparison, the effect of the Au modification was better than that of Ag. For Ti_3_C_2_F_2_, the Ag and Au doping modifications did not significantly change the adsorption effects for HCHO and C_6_H_6_. However, the Ag and Au doping modifications decreased the adsorption of Ti_3_C_2_(OH)_2_ for HCHO, while there was no significant change in the gas adsorption for C_6_H_6_. The above results serve as a theoretical foundation for the design of new sensors for HCHO and C_6_H_6_.

## 1. Introduction

With the rapid growth in the economy, the production and sale of cars have gradually increased, becoming one of the most important tools for people’s travel. The carriage is a special, enclosed environment with a wide variety of facilities in a limited space [1,2]. In enclosed cars, some volatile organic compounds (VOCs) are commonly present, especially with new cars and interior decorations made of leather materials, for which the concentration of VOCs is significantly higher [3,4,5]. VOCs not only produce abnormal odors and affect comfort but also have the potential to affect the health of drivers and passengers [5]. Among them, formaldehyde (HCHO) and benzene (C_6_H_6_) are two VOCs with higher levels of content [6]. HCHO is an irritant with damaging effects on the respiratory tract, skin, and eyes, which has been listed as a carcinogen and teratogen by the World Health Organization [7,8]. C_6_H_6_ can cause nerve center paralysis, dizziness, nausea, insomnia, and other symptoms, and it has also been identified as a strong carcinogen [9,10]. Even if their concentrations are not high inside a carriage, prolonged exposure can lead to chronic poisoning. Therefore, in order to ensure human health, it is necessary to monitor the concentrations of HCHO and C_6_H_6_ inside cars.

Gas sensors are widely used in online gas monitoring because of their small volume, fast detection speed, and low cost [11]. In recent years, researchers have continuously explored gas-sensing materials [12,13]. Extensive research has been conducted on intrinsic graphene and metal-oxide-modified graphene, revealing their superior gas sensitivity to SF_6_ decomposition gases, nitrogen oxides, and other gases [14,15,16,17,18]. Subsequently, adsorption studies of two-dimensional transition metal disulfides (TMDs) on SF_6_ decomposition gas, dissolved gas in transformer oil, nitrogen oxides, etc., have also been extensively carried out [19,20,21,22]. At present, research on adsorption materials for VOCs mainly focuses on MoS_2_, C_2_N, SiC, and other materials. However, existing research indicates that continual search for and exploration of suitable materials is still needed. MXene, as a 2D transition metal carbide/carbon nitride, has a unique morphology; superior optical, electrical, and catalytic properties: and has been widely studied since its discovery in 2011 [23,24,25,26]. Among them, Ti_3_C_2_T_x_ is the first synthesized and widely used MXene [27,28]. Wang et al.’s study shows that single-layer Ti_3_C_2_T_x_ can remove Cr (VI) from wastewater [29]. Related studies show that Ti_3_C_2_T_x_ has a stable structure and strong hydrogen storage capacity, which makes it a potential candidate for sensing VOCs [30]. Alfalasi et al. investigated the adsorption performance of Ti_3_C_2_T_x_ on six types of VOCs [31]. Zhou et al. used a Ti-modified Ti_3_C_2_O_2_ monolayer (MXene) for formaldehyde oxidation [32]. All of these studies show that Ti_3_C_2_T_x_ has excellent potential for VOCs adsorption. Yao et al. fabricated a nanodiamond (ND)/Ti3C2 MXene composite-coated QCM humidity sensor [33]. The great potential of PEDOT:PSS/MXene composite sensors for room-temperature NH_3_ detection was experimentally verified by Jin et al. [34]. This also illustrates the reliability of MXene materials in practical applications. Moreover, numerous studies show that the selection of suitable doped atoms or molecules can enhance the gas-sensing properties of intrinsic materials [16,22]. Metal atoms are commonly used for material surface modifications. Among them, Ag and Au are often chosen due to their low oxidation resistance. Zeng et al. used Au-modified Ti_3_C_2_T_x_ for the adsorption of SF_6_/N_2_-mixed decomposition gases and confirmed its good sensing ability for NO and NO_2_ [35]. Nan et al. used Au- and Pt-modified Ti_3_C_2_T_x_ MXene gas sensors for sensing NH_3_ [36]. Wu et al. studied the room-temperature NH_3_-sensing properties and humidity influence of Ti_3_C_2_T_x_ and Ag-Ti_3_C_2_T_x_ in an oxygen-free environment [37]. The above studies are sufficient to demonstrate the applicability of modifications of Ag atoms and Au atoms on Ti_3_C_2_T_x_ monolayers, which is why Ag atoms and Au were selected for this paper.

This study selected Ti_3_C_2_T_x_ as the substrate material and Ag and Au metal atoms as modified atoms to construct a stable TM-Ti_3_C_2_T_x_ (TM=Ag, Au) structure. Then, the gas-sensing performance of Ag-/Au-modified Ti_3_C_2_T_x_ for HCHO and C_6_H_6_ gas molecules was studied by analyzing the adsorption energy, adsorption distance, charge transfer, charge deformation density (CDD), total density of states (TDOS), and partial density of states (PDOS). This study lays a theoretical foundation for the design of new sensors for HCHO and C_6_H_6_.

## 2. Results and Discussion

### 2.1. Molecular Structure and Adsorption Properties of Intrinsic Ti_3_C_2_T_x_

#### 2.1.1. Structures of Ti_3_C_2_T_x_, C_6_H_6_, and HCHO

**Figure 1:**
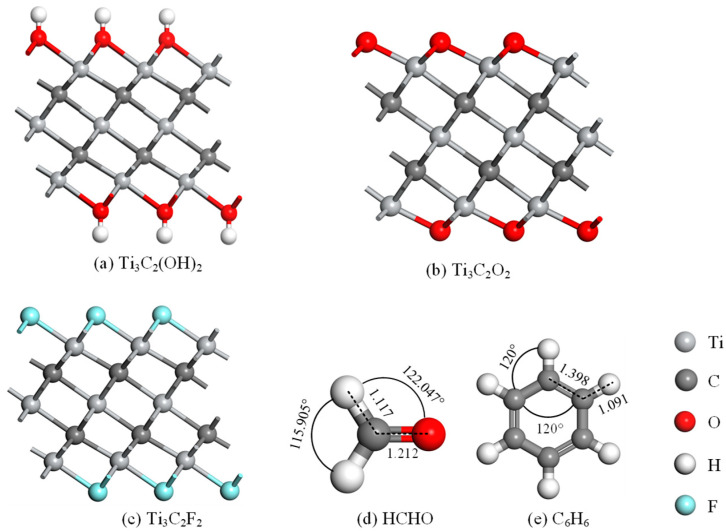
Structures of (**a**) HCHO; (**b**) C_6_H_6_; (**c**) Ti_3_C_2_O_2_; (**d**) Ti_3_C_2_F_2_; (**e**) Ti_3_C_2_(OH)_2_. Distances are in Å.

To clarify the structural changes during the processes of modification and adsorption, the optimized structures of the intrinsic Ti_3_C_2_T_x_ and gas molecules were investigated. As shown in Figure 1, the structures of Ti_3_C_2_O_2_, Ti_3_C_2_F_2_, and Ti_3_C_2_(OH)_2_ are similar, with alternating connections between Ti and C in the middle of the molecular layer, presenting an interlayer structure of Ti-C. The Ti atoms on the upper and lower surfaces are connected to the -O, -F, and -OH functional groups, which stabilizes the structure. Both HCHO and C_6_H_6_ gas molecules have two-dimensional planar structures. The HCHO molecule is centered on the C atom, with a C-H bond length of 1.117 Å and a C-O bond length of 1.212 Å. The angles of H-C-H and H-C-O are 115.905° and 122.047°, respectively. The C_6_H_6_ molecule has a regular hexagonal structure with C-C-C and C-C-H angles of 120°, C-C bond length of 1.398 Å, and C-H bond length of 1.091 Å.

#### 2.1.2. Adsorption Performance of Intrinsic Ti_3_C_2_T_x_ for C_6_H_6_ and HCHO

**Figure 2:**
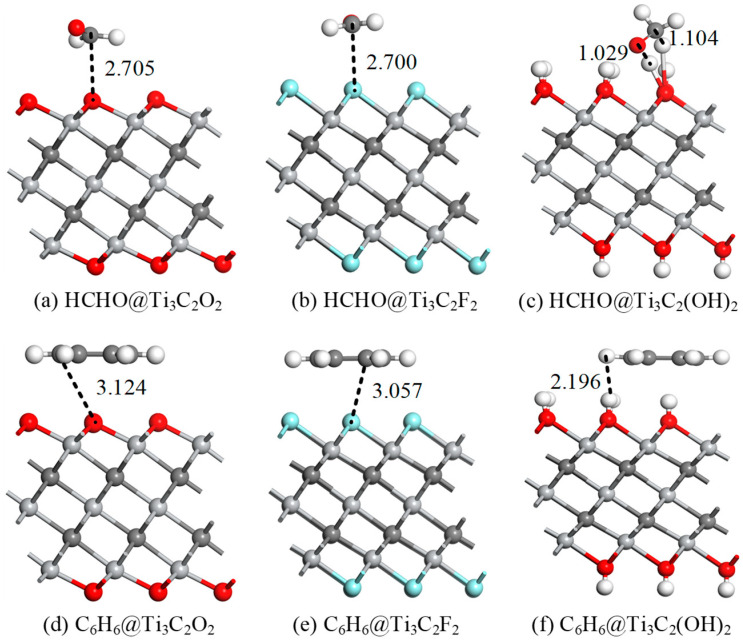
The adsorption structures of HCHO and C_6_H_6_ gases on pristine Ti_3_C_2_T_x_. Distance are in Å.

Figure 2 shows the stable adsorption structures of HCHO and C_6_H_6_ on intrinsic Ti_3_C_2_T_x_, and the corresponding data are listed in Table 1. The adsorption energies of HCHO on Ti_3_C_2_O_2_ and Ti_3_C_2_F_2_ are −0.425 eV and −0.347 eV, respectively, with adsorption distances of 2.705 Å and 2.7 Å, indicating that the adsorption effects are weak. This is mainly due to the insufficient force of the -O and -F functional groups on the HCHO molecules. Although the adsorption energies of C_6_H_6_ on Ti_3_C_2_O_2_ and Ti_3_C_2_F_2_ reach −1.298 eV and −1.108 eV, the adsorption distances on both substrates exceed 3 Å. Therefore, the adsorption performance still needs to be improved. The adsorption energies of HCHO and C_6_H_6_ on Ti_3_C_2_(OH)_2_ reach −3.665 eV and −1.444 eV, respectively, and the adsorption distances are reduced to 1.029 Å and 2.196 Å, demonstrating excellent gas-sensing properties. The Hirshfeld method indicates that the charge transfer amounts of HCHO adsorbed on intrinsic Ti_3_C_2_T_x_ are 0.058 *e*, −0.027 *e*, and −0.223 *e*, while the charge transfer amounts of C_6_H_6_ adsorbed on intrinsic Ti_3_C_2_T_x_ are 0.075 *e*, −0.030 *e*, and −0.180 *e*, respectively. For gas adsorption on Ti_3_C_2_O_2_, electrons are transferred from the HCHO and C_6_H_6_ gas molecules to the substrate, while for gas adsorption on Ti_3_C_2_F_2_ and Ti_3_C_2_(OH)_2_, electrons are transferred from the substrates to the HCHO and C_6_H_6_ gas molecules. This difference mainly stems from the varying abilities of the three functional groups—-O, -F, and -OH—to gain and lose electrons. The above analysis shows that Ti_3_C_2_O_2_ and Ti_3_C_2_F_2_ have limited gas sensitivities to HCHO molecules and C_6_H_6_ molecules, which should be modified to improve the adsorption capacity. However, Ti_3_C_2_(OH)_2_ has a strong adsorption capacity for the two gases, so the improvement effect of the modification on the adsorption performance may not be obvious.

### 2.2. Modification of Ti_3_C_2_T_x_ with Ag and Au

**Figure 3:**
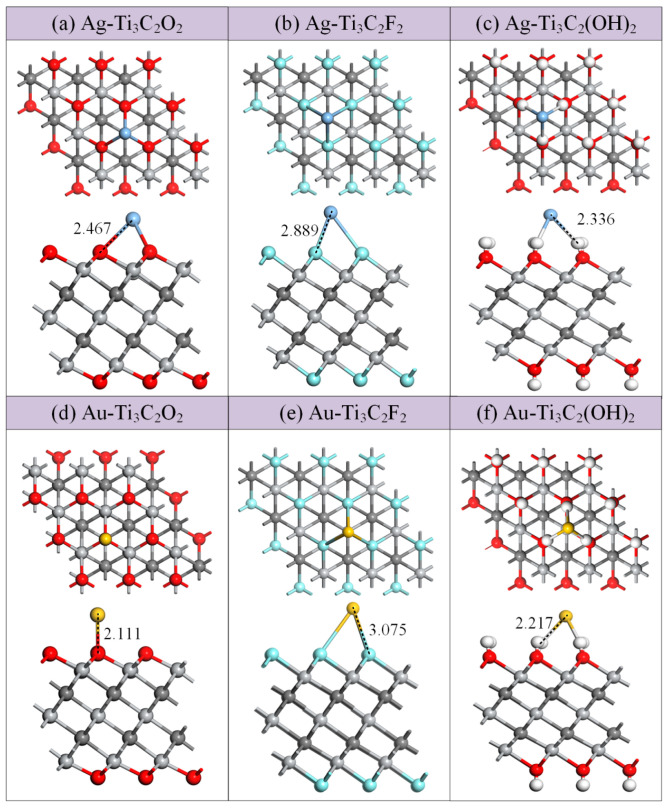
Top and side views: (**a**) Ag-Ti_3_C_2_O_2_; (**b**) Ag-Ti_3_C_2_F_2_; (**c**) Ag-Ti_3_C_2_(OH)_2_; (**d**) Au-Ti_3_C_2_O_2_; (**e**) Au-Ti_3_C_2_F_2_; (**f**) Au-Ti_3_C_2_(OH)_2_. Distances are in Å.

It can clearly be seen from the top view of the Ti_3_C_2_T_x_ that the modification with metal atoms occurs at three doping sites, namely, the top of the Ti atom, the top of the C atom, and the top of the T functional group. Firstly, the modified structures formed by the differently doped sites were optimized, and then the structure with the highest absolute value of the binding energy was selected as the stable structure, as shown in Figure 3, which was used for subsequent research.

From Figure 3, it can be seen that the modification with Ag and Au atoms did not cause significant changes in the structure of Ti_3_C_2_T_x_, which is beneficial for the stability of the modified structure. Among these, only the metal atom in Au-Ti_3_C_2_O_2_ is connected to a single functional group, -O, of the substrate, while the metal atoms in the other modified structures are connected to the three functional groups of the substrate. Table 2 presents the corresponding parameters of the binding energies, binding distances, and charge transfer amounts of these modified structures. Among them, Ag-Ti_3_C_2_O_2_ is formed by Ag located at the top of the Ti atom, while Ag-Ti_3_C_2_F_2_ and Ag-Ti_3_C_2_(OH)_2_ are formed by Ag located at the top of the C atom, with binding energies of −1.879 eV, −0.635 eV, and −2.787 eV, respectively. The closest distances between the Ag atoms and the Ti_3_C_2_O_2_, Ti_3_C_2_F_2_, and Ti_3_C_2_(OH)_2_ surfaces are 2.467 Å (Ag-O), 2.889 Å (Ag-F), and 2.368 Å (Ag-H), respectively. During the modification process, electrons of 0.5366 *e* and 0.2393 *e* are transferred from the Ag atom to the Ti_3_C_2_O_2_ and Ti_3_C_2_F_2_ surfaces, respectively, while electrons of 0.3141 *e* are transferred from the Ti_3_C_2_(OH)_2_ surface to the Ag atom. For the modification with Au atoms, Au-Ti_3_C_2_O_2_ is formed by Au located at the top of the O atom, while Au-Ti_3_C_2_F_2_ and Au-Ti_3_C_2_(OH)_2_ are formed by Au located at the top of the Ti atom, with binding energies of −1.103 eV, −0.484 eV, and −3.950 eV, respectively. The closest distances between the Au atoms and Ti_3_C_2_O_2_, Ti_3_C_2_F_2_, and Ti_3_C_2_(OH)_2_ surfaces are 2.111 Å (Au-O), 3.130 Å (Au-F), and 2.217 Å (Au-H), respectively. During the modification process, electrons of 0.297 *e* are transferred from the Ag atom to the Ti_3_C_2_O_2_ surface, while electrons of 0.031 *e* and 0.331 *e* are transferred from the Ti_3_C_2_F_2_ and Ti_3_C_2_(OH)_2_ surfaces to the Au atom, respectively. Among the six modified structures, it is obvious that the absolute binding energies and charge transfers of the two modified structures of Ti_3_C_2_F_2_ are relatively small and the adsorption distance is relatively large. So the modified structure of Ti_3_C_2_F_2_ is less stable than that of Ti_3_C_2_O_2_ and Ti_3_C_2_(OH)_2_. Overall, the negative binding energies indicate that the modifications of Ti_3_C_2_T_x_ with Ag and Au atoms are spontaneous.

**Figure 4:**
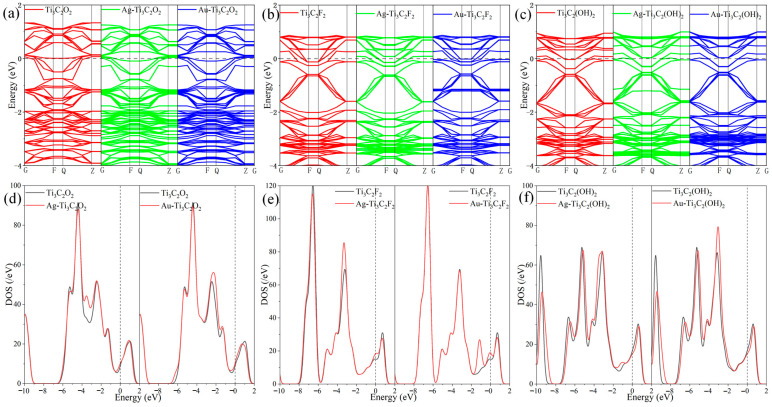
(**a**–**c**) The band structures and (**d**–**f**) TDOS of Ti_3_C_2_T_x_ and TM-Ti_3_C_2_T_x_.

Figure 4 shows the band structures and TDOS of the systems before and after metal atom modification. In Figure 4a–c, the red curves represent the band structures of the intrinsic Ti_3_C_2_T_x_, the green curves represent the band structures of the system after Ag atom modification, and the blue curves represent the band structures of the system after Au atom modification. After the modifications with Ag and Au atoms, the band distributions were denser, but these changes are mainly reflected in the valence bands below the Fermi level, indicating that the changes in the system’s conductivity were not significant. In Figure 4d–f, the black curves represent the TDOS distributions of the intrinsic Ti_3_C_2_T_x_, and the red curves represent the TDOS distributions of the modified structures. A comparison of the black and red curves shows that the TDOS from −4 eV to −3 eV or from −2.3 eV to −1.7 eV of Ti_3_C_2_O_2_ increased after the modification with the Ag or Au atom, respectively, while the modifications with Ag and Au increased the TDOS of Ti_3_C_2_F_2_ near the Fermi level, resulting in an increase in the conductivity of the systems. For Ti_3_C_2_(OH)_2_, the modified TDOS curve shows a slight right shift, causing more electrons to fill the conduction band. Overall, modifications with Ag and Au may lead to an increase in the conductivity of Ti_3_C_2_T_x_.

### 2.3. Adsorption Performance of HCHO on TM-Ti_3_C_2_T_x_

**Figure 5:**
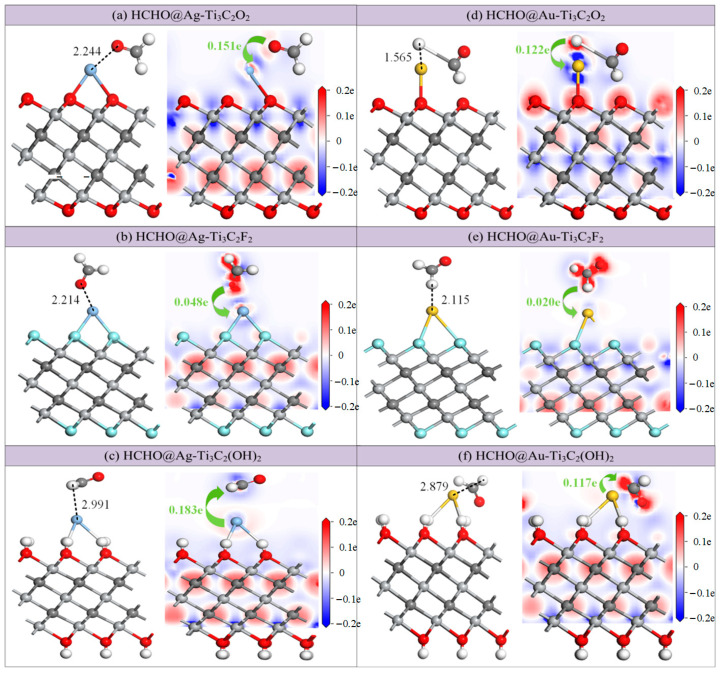
Stable structures and CDDs of HCHO adsorption on (**a**) Ag-Ti_3_C_2_O_2_; (**b**) Ag-Ti_3_C_2_F_2_; (**c**) Ag-Ti_3_C_2_(OH)_2_; (**d**) Au-Ti_3_C_2_O_2_; (**e**) Au-Ti_3_C_2_F_2_; (**f**) Au-Ti_3_C_2_(OH)_2_. Distances are in Å.

The adsorption structures of HCHO molecules closed to the Ag and Au atoms on the Ti_3_C_2_T_x_ at different initial positions were studied. The one with the highest adsorption energy was selected for detailed analysis. Figure 5 shows the stable structures of these adsorption systems, and a comparison of the parameters between the modified adsorption and intrinsic adsorption are presented in Table 3. After modification with Ag or Au, the adsorption energies of Ti_3_C_2_O_2_ for HCHO increase to −1.081 eV and −1.460 eV, respectively, and the adsorption distances are shortened to 2.244 Å and 1.565 Å, while the charge transfer amounts increase to 0.1513 e and 0.1219 e. However, after adsorption on Au-Ti_3_C_2_O_2_, HCHO undergoes severe deformation, with one C-H bond severely stretched from 1.117 Å to 2.579 Å, which is not conducive to the gas desorption process. Therefore, Au-Ti_3_C_2_O_2_ is suitable for the adsorption of HCHO but not for the sensing of HCHO. On the modified Ti_3_C_2_F_2_ surface, although the distances between HCHO and the Ag/-Au-modified Ti_3_C_2_T_x_ are shortened from 2.7 Å to 2.214 Å and 2.115 Å, the adsorption energies remain at only −0.489 eV and −0.339 eV. Although the charge transfers change from negative to positive, the absolute values of the changes are very small, indicating that the modifications with Ag and Au do not have a significant effect on enhancing the gas sensitivity of Ti_3_C_2_F_2_ to HCHO. The intrinsic Ti_3_C_2_(OH)_2_ has an excellent gas sensitivity to HCHO, but after modification with Ag and Au, the adsorption performance of HCHO actually decreases. The adsorption energies of Ag-Ti_3_C_2_(OH)_2_ and Au-Ti_3_C_2_(OH)_2_ for HCHO decrease significantly, from −3.665 eV to −0.303 eV and −0.951 eV, and the adsorption distances are extended from 1.029 Å to 2.991 Å and 2.898 Å, with charge transfer amounts of −0.1833 *e* and −0.1172 *e*. This indicates that the doping with Ag and Au atoms disrupts the unique force of the -OH functional group on HCHO.

The right columns in Figure 5a–f show the CDDs of the HCHO adsorption structure, which can be used to further investigate the changes in electron distribution during the adsorption process. The atomic charge density in the red area increases, while the atomic charge density in the blue area decreases, and the depth of the color represents the degree of change. Affected by the vacuum layer setting area, the CDD of the lower part of all adsorption structures is not displayed. It can be seen that the C, O, and F atoms on the substrate always lose electrons and the charge density increases, while the Ti atom acts as an electron acceptor and the charge density decreases. Due to the strong electronegativity of the O and F atoms, coupled with the interaction of the Ag and Au atoms, electrons always transfer from the HCHO molecules to the modified Ti_3_C_2_O_2_ and Ti_3_C_2_F_2_ substrates. For the Ti_3_C_2_(OH)_2_ adsorption systems, because of the electron loss ability of H atoms, electrons transfer from the substrate to the gas molecules.

**Figure 6:**
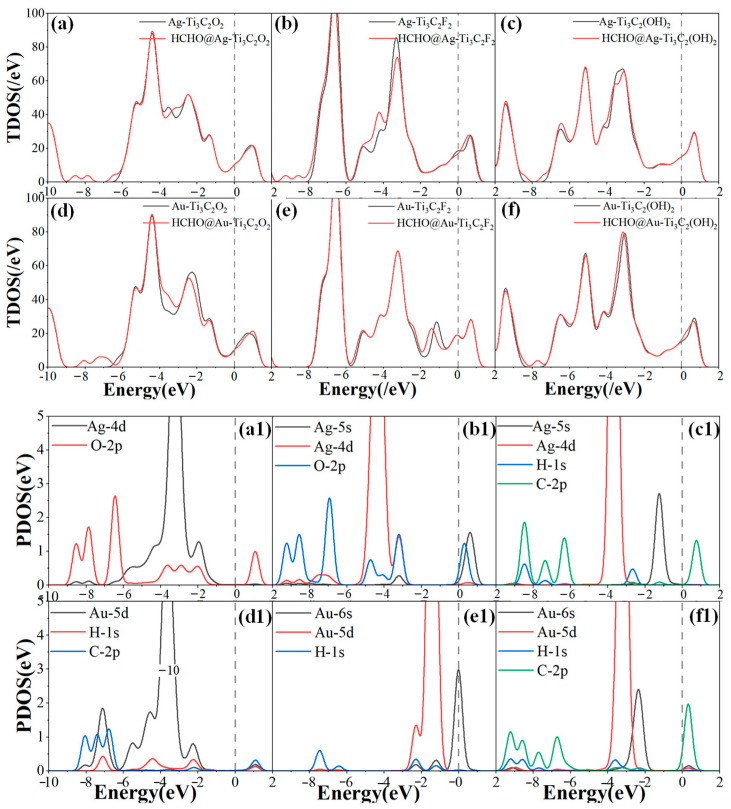
TDOS and PDOS of HCHO adsorption on (**a**,**a1**) Ag-Ti_3_C_2_O_2_; (**b**,**b1**) Ag-Ti_3_C_2_F_2_; (**c**,**c1**) Ag-Ti_3_C_2_(OH)_2_; (**d**,**d1**) Au-Ti_3_C_2_O_2_; (**e**,**e1**) Au-Ti_3_C_2_F_2_; (**f**,**f1**) Au-Ti_3_C_2_(OH)_2_.

Figure 6a–f show the TDOS of the modified structures before and after adsorption. While Figure 6(a1–f1) show the PDOS of the modified adsorption structures, where C, H, and O atoms represent the atoms of the HCHO gas molecule. After Ag-Ti_3_C_2_O_2_ adsorbs HCHO, the Ag–4d orbitals and O–2p orbitals exhibit orbital hybridization at −8.5 eV, −7.8 eV, and −2 eV, which also leads to an increase in TDOS at the corresponding energy levels. Figure 6d shows that after HCHO adsorption on Ag-Ti_3_C_2_F_2_, there is an increase in the TDOS of between −8.5 eV and −7.5 eV, as well as between −4 eV and −3 eV. In Figure 6(d1), the PDOS curves of H–1s and Au–5d orbitals overlap at −7 eV, while the Au–5d, H–1s, and C–2p orbitals overlap at −2.2 eV and 1.1eV, verifying the strong interaction force between Au and H. After Ag-Ti_3_C_2_F_2_ and Au-Ti_3_C_2_F_2_ adsorb HCHO, there are changes in the TDOS of between −5 eV and −3 eV, as well as between −2 eV and −0.5 eV, respectively. There is only weak hybridization between Ag–4d orbitals and O–2p orbitals, as well as between Au–5d orbitals and H–1s orbitals. It also shows that the adsorption capacity of Ag-Ti_3_C_2_F_2_ and Au-Ti_3_C_2_F_2_ for HCHO is relatively weak. Figure 6c,f indicate that the TDOS curves only show slight changes locally before and after HCHO adsorption. At the same time, Figure 6(c1,f1) also show that there is no significant hybridization between metal atoms and gas molecules, which verifies the weak adsorption ability of Ag-Ti_3_C_2_(OH)_2_ and Au-Ti_3_C_2_(OH)_2_ for HCHO. This is consistent with previous research on adsorption energy, adsorption distance, etc. Overall, the modifications with Ag and Au improved the adsorption performance of Ti_3_C_2_O_2_ for HCHO but had no significant effect on that of Ti_3_C_2_F_2_ while reducing the gas sensitivity of Ti_3_C_2_(OH)_2_ to HCHO.

### 2.4. Adsorption Performance of C_6_H_6_ on TM-Ti_3_C_2_T_x_

**Figure 7:**
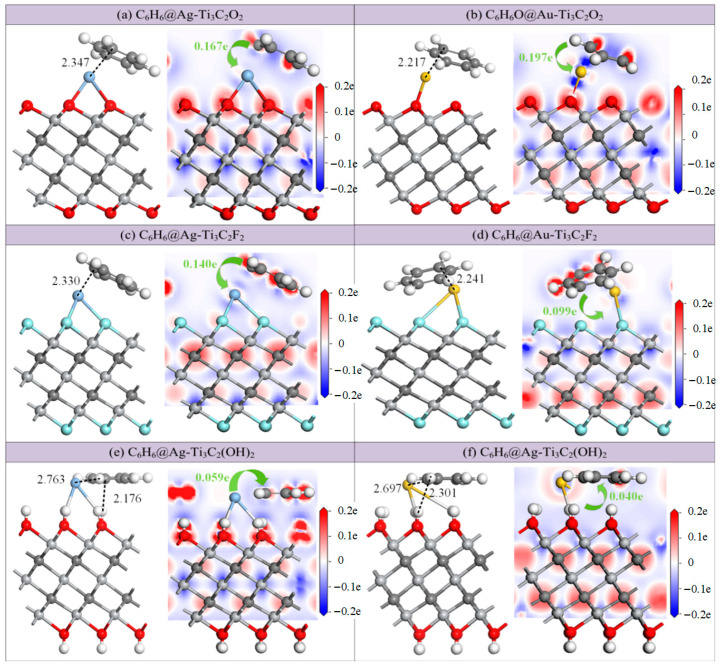
Stable structures and CDDs of C_6_H_6_ adsorption on (**a**) Ag-Ti_3_C_2_O_2_; (**b**) Ag-Ti_3_C_2_F_2_; (**c**) Ag-Ti_3_C_2_(OH)_2_; (**d**) Au-Ti_3_C_2_O_2_; (**e**) Au-Ti_3_C_2_F_2_; (**f**) Au-Ti_3_C_2_(OH)_2_. Distances are in Å.

Similarly, by placing C_6_H_6_ at different initial positions above the modified substrate, the structure with the highest adsorption energy was selected, as shown in Figure 7. Table 4 presents the adsorption parameters before and after modification for a comparative analysis of the improvement effect of the Ag and Au atoms. After the modifications with Ag and Au atoms, the adsorption energies of Ti_3_C_2_O_2_ for C_6_H_6_ increased from −1.298 eV to −1.852 eV and −2.467 eV, with adsorption distances of 2.347 Å between the Ag and C atoms and 2.217 Å between the Au and C atoms. The charge transfer amounts increase by more than twice, to 0.167 *e* and 0.197 *e*. Compared with the intrinsic Ti_3_C_2_F_2_, the adsorption energies of Ag-Ti_3_C_2_F_2_ and Au-Ti_3_C_2_F_2_ for C_6_H_6_ do not change significantly, with values of −1.297 eV and −1.211 eV, respectively. The adsorption distances decrease to 2.330 Å and 2.241 Å, and the charge transfer amounts change from −0.003 *e* to 0.140 *e* and 0.099 *e*. These weak changes indicate that the modification effects of the Ag and Au atoms on Ti_3_C_2_F_2_ are not satisfactory. For the Ti_3_C_2_(OH)_2_ substrate, the adsorption energies of C_6_H_6_ slightly decrease after modification, reaching −1.395 eV and −1.431 eV, and the charge transfer amounts also decrease from −0.180 *e* to −0.059 *e* and −0.040 *e*. On the surfaces of the Ag-Ti_3_C_2_(OH)_2_ and Au-Ti_3_C_2_(OH)_2_, the distances between the Ag and Au atoms and the gas molecules are actually greater than the distances between the H atoms on the surfaces of the Ti_3_C_2_(OH)_2_ and gases. This indicates that the Ag and Au atom modifications did not enhance the gas sensitivity of the intrinsic Ti_3_C_2_(OH)_2_ for C_6_H_6_.

In order to further investigate the changes in electron distribution during the adsorption process, the CDDs of all adsorption structures were analyzed, as shown in the right columns of Figure 7a–f. The O, C, and F atoms on the substrates are red, indicating the loss of electrons and an increase in the charge density. The Ag, Au, and Ti atoms appear blue, indicating the acquirement of electrons and a decrease in the charge density. Due to the electron loss properties of both the C and H atoms in C_6_H_6_, except for the modified structure of Ti_3_C_2_(OH)_2_, electrons are always transferred from the C_6_H_6_ gas molecule to the modified substrate, while electrons are transferred from the surfaces of the Ag-Ti_3_C_2_(OH)_2_ and Au-Ti_3_C_2_(OH)_2_ to the C_6_H_6_ gases; this is mainly due to the electron loss ability of the H atoms on the substrate.

**Figure 8:**
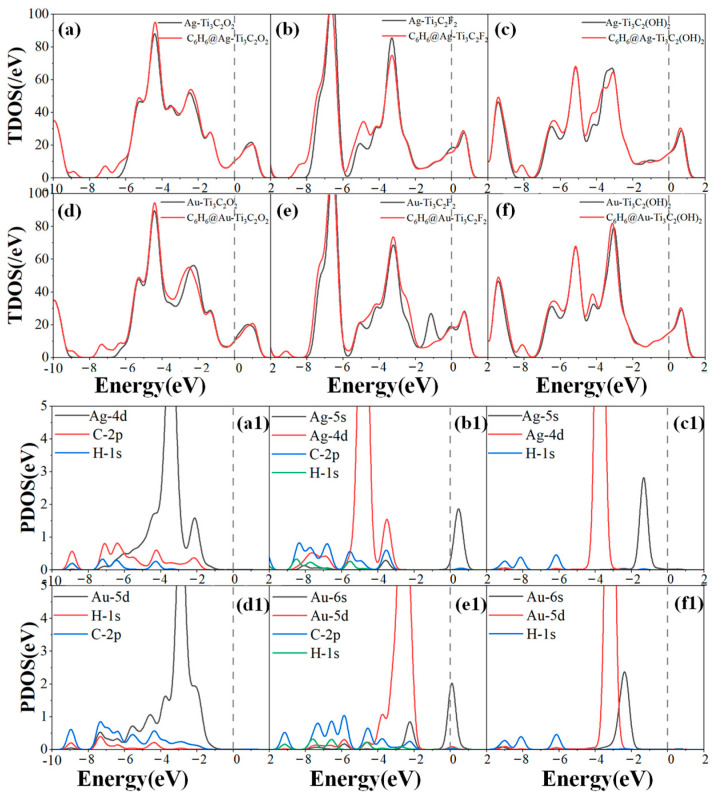
TDOS and PDOS of C_6_H_6_ adsorption on (**a**,**a1**) Ag-Ti_3_C_2_O_2_; (**b**,**b1**) Ag-Ti_3_C_2_F_2_; (**c**,**c1**) Ag-Ti_3_C_2_(OH)_2_; (**d**,**d1**) Au-Ti_3_C_2_O_2_; (**e**,**e1**) Au-Ti_3_C_2_F_2_; (**f**,**f1**) Au-Ti_3_C_2_(OH)_2_.

Similarly, Figure 8a–f show the TDOS before and after adsorption of the modified structure, while Figure 8(a1–f1) show the PODS of the modified adsorption structure, also the C, H, and O atoms are from the HCHO gas molecule. After adsorbing C_6_H_6_, the TDOS of Ag-Ti_3_C_2_O_2_ increased significantly to around −9 eV, −7 eV, and −4.5 eV, mainly due to the hybridization between the atoms of the gas molecule itself. Figure 8(d1) shows that the increase in the TDOS of Au-Ti_3_C_2_O_2_ after the adsorption of C_6_H_6_ is mainly due to the hybridization of Au–5d orbitals with H–1s orbitals and C–2p orbitals at −9 eV, −7.5 eV, and −5.5 eV. After adsorbing C_6_H_6_, Ag-Ti_3_C_2_F_2_ showed a significant increase in TDOS between −9 eV and −7 eV, as well as between −6 eV and −4eV, while a decrease was observed at −3.2 eV and Fermi levels, indicating a decrease in system conductivity. In Figure 8(b1), the Ag–4d, Ag–5s orbitals, and C–2p orbitals only exhibited hybridization at −3.5 eV. The TDOS of the C_6_H_6_@Au-Ti_3_C_2_F_2_ system shows a peak removal decrease between −2 eV and −0.7 eV compared to that of Au-Ti_3_C_2_F_2_. Figure 8(e1) shows weaker hybridizations of the Au–5d and Au–6s orbitals with the C–2p and H–1s orbitals. After adsorbing C_6_H_6_, the overall TDOS of Ag-Ti_3_C_2_(OH)_2_ and Au-Ti_3_C_2_(OH)_2_ show increasing trends, mainly caused by the C_6_H_6_ gas molecule itself, as there was no hybridization effect between the Ag and Au metal atoms and gas molecules, as shown in Figure 8(c1,f1). In summary, the modifications with Ag and Au had significant effects on enhancing the gas sensitivity of Ti_3_C_2_O_2_ to C_6_H_6_, with a weak effect on Ti_3_C_2_F_2_ and almost no effect on Ti_3_C_2_(OH)_2_.

### 2.5. Comparison of Adsorption Performances of HCHO and C_6_H_6_ in Different Systems

**Figure 9:**
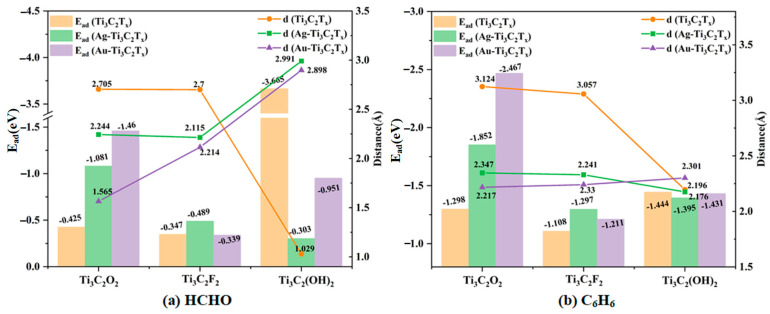
Adsorption energies and distances of (**a**) HCHO and (**b**) C_6_H_6_ on Ti_3_C_2_T_x_ and MO-Ti_3_C_2_T_x_.

In order to visually reflect the improvement effect of the Ag and Au atomic modification on the gas sensitivity characteristics of Ti_3_C_2_T_x_, bar charts and dot plots are provided in Figure 9, with the adsorption data of HCHO in Figure 9a and the adsorption data of C_6_H_6_ in Figure 9b. For Ti_3_C_2_O_2_, after the modifications with the Ag and Au substrates, the adsorption energies of HCHO and C_6_H_6_ by the substrates were greatly improved, and the adsorption distances also decreased significantly. This indicates that the modification with Ag and Au significantly improved the gas sensitivity of Ti_3_C_2_O_2_ for the two gases. However, after the modification of Ti_3_C_2_F_2_, although the adsorption distances of the two gases decreased, the adsorption energies changed weakly, so the modification effects of Ag and Au were not obvious. Similarly, the adsorption energy and adsorption distance for C_6_H_6_ of Ti_3_C_2_(OH)_2_ changed weakly after modification, so the modification effect was also weak. On the contrary, the adsorption energy of MO-Ti_3_C_2_(OH)_2_ for HCHO seriously decreased, and the adsorption distance greatly increased, indicating that the good gas sensitivity of Ti_3_C_2_(OH)_2_ was destroyed. In conclusion, the modifications with Ag and Au only had positive effects on the Ti_3_C_2_O_2_ monolayer. In addition, according to the values of the adsorption energies, the best adsorption effects for the two gases were Au-Ti_3_C_2_O_2_, Ag-Ti_3_C_2_F_2_, and Ti_3_C_2_(OH)_2_ base.

The good adsorption materials obtained by previous researchers were selected and compared with the results of this study, and the data are listed in Table 5. It can be seen that for HCHO, the Ti_3_C_2_(OH)_2_ material proposed here exhibits an excellent performance, with a greater adsorption energy compared to the previous research materials. For the adsorption of C_6_H_6_ gas molecules, the proposed Au-Ti_3_C_2_O_2_ material is superior to the previously studied materials. However, other materials obtained in this paper exhibit no breakthrough compared with previous studies. According to the research in this field, for some materials, multiple-atom doping leads to a more obvious improvement effect than single-atom doping. Therefore, for the Ti_3_C_2_T_x_ monolayer, the gas sensitivity can be further studied by increasing the doping number of Ag and Au atoms in the future.

## 3. Methods

All calculations in this study are based on DFT in Dmol^3^. The calculation model used a 4 × 4 × 1 supercell of Ti_3_C_2_T_x_ as the substrate, and in order to prevent mutual influence between adjacent layers, a 20 Å vacuum layer was set, using the generalized approximation (GGA) method with the PerdewBurke–Ernzerhof (PBE) function to calculate the interaction-related energy [41,42]. Tkatchenko and Scheffler’s (TS) method was used to understand the Van der Waals’ force in the calculations. The DFT semi-core pseudopots (DSSP) method and double numerical plus polarization (DNP) were chosen as the atomic orbital basis set [43]. The energy tolerance accuracy, maximum force, and displacement were 10^−5^ Ha, 2 × 10^−3^ Ha/Å, and 5 × 10^−3^ Å, respectively [43]. To ensure the accuracy of the total energy in the calculation of the static electronic structure, the convergence accuracy of the SCF was set to 10^−6^ Ha.

The binding energy (*E_b_*) represents the change in the system’s energy during the process of Ag and Au modification on Ti_3_C_2_T_x_, which can be defined by Equation (1), where *E_TM-Ti3C2Tx_*, *E_Ti3C2Tx_*, and *E_Mo_* represent the energy of the modified system, Ti_3_C_2_T_x_ monolayer, and Mo (M=Ag, Au) single atom, respectively. Similarly, the adsorption energy (*E_ad_*) is calculated by Equation (2), where *E_surf_* and *E_gas_*_/*surf*_ represent the energy of the system before and after gas adsorption, and *E_gas_* represents the energy of a single gas molecule. If *E_b_* and *E_ad_* are negative, the reaction releases heat and can proceed spontaneously. The charge transfer (*Q_t_*), calculated by Equation (3), was analyzed by the Hirshfeld method [44], where *Q_ads_* represents the total load of gas molecules after adsorption, and *Q_iso_* represents the total load of isolated gas. For gas molecules, a positive *Q_t_* value represents the loss of electrons.

(1) $$E_{b}=E_{Mo-Ti_{3}C_{2}T_{x}}-E_{Ti_{3}C_{2}T_{x}}-E_{Mo}$$

(2) $$E_{ad}=E_{gas/surf}-E_{surf}-E_{gas}$$

(3) $$Q_{t}=Q_{ads}-Q_{iso}$$

## 4. Conclusions

Based on DFT calculations, this study analyzed the adsorption performances of HCHO and C_6_H_6_ gases on Ag- and Au-modified Ti_3_C_2_T_x_ (T=O, F, OH) by analyzing the adsorption energy, adsorption distance, charge transfer amount, CDD, TDOS, and PDOS. The modification processes of Ti_3_C_2_T_x_ by Ag and Au occur spontaneously, and the modified structures are stable. Ag-Ti_3_C_2_O_2_ and Au-Ti_3_C_2_O_2_ have better adsorption performances for HCHO and C_6_H_6_ than Ti_3_C_2_O_2_. In comparison, the modification effect of Au is better than that of Ag. For Ti_3_C_2_F_2_, Ag and Au doping modifications do not significantly enhance the adsorption effects for HCHO and C_6_H_6_. However, Ag and Au doping modifications decrease the adsorption of Ti_3_C_2_(OH)_2_ for HCHO, while there is no significant change in the adsorption for C_6_H_6_. Under combination, the best adsorption material for HCHO obtained in this study was Ti_3_C_2_(OH)_2_, with an adsorption energy of −3.665 eV, while the best adsorption material for C_6_H_6_ was Au-Ti_3_C_2_O_2_, with an adsorption energy of −2.467 eV. Thus, this study presents potential and efficient adsorbed materials for HCHO and C_6_H_6_.

## Figures and Tables

**Table 1:** Parameters of the most stable adsorption structures of the gas molecules on an intrinsic Ti_3_C_2_T_x_ surface.

Gas	Structure	*E*_ad_ (eV)	Distance (Å)	*Q_t_* (*e*)
HCHO	Ti_3_C_2_O_2_	−0.425	2.705 (C-O)	0.058
	Ti_3_C_2_F_2_	−0.347	2.700 (C-F)	−0.027
	Ti_3_C_2_(OH)_2_	−3.665	1.029 (O-H)	−0.223
C_6_H_6_	Ti_3_C_2_O_2_	−1.298	3.124 (C-O)	0.075
	Ti_3_C_2_F_2_	−1.108	3.057 (C-F)	−0.030
	Ti_3_C_2_(OH)_2_	−1.444	2.196 (H-H)	−0.180

**Table 2:** Parameters of the Ag- and Au-modified Ti_3_C_2_T_x_ monolayers.

Structure	*E*_b_ (eV)	Distance (Å)	*Q_t_* (*e*)
Ag-Ti_3_C_2_O_2_	−1.879	2.467 (Ag-O)	0.534
Ag-Ti_3_C_2_F_2_	−0.635	2.889 (Ag-F)	0.239
Ag-Ti_3_C_2_(OH)_2_	−2.787	2.366 (Ag-H)	−0.314
Au-Ti_3_C_2_O_2_	−1.103	2.111 (Au-O)	0.297
Au-Ti_3_C_2_F_2_	−0.484	3.075 (Au-F)	−0.031
Au-Ti_3_C_2_(OH)_2_	−3.950	2.217 (Au-H)	−0.331

**Table 3:** Comparative parameters of HCHO adsorption on the intrinsic and modified Ti_3_C_2_T_x_ surfaces.

Structure	*E*_ad_ (eV)	Distance (Å)	*Q_t_* (*e*)
Ti_3_C_2_O_2_	−0.425	2.705 (C-O)	0.058
Ag-Ti_3_C_2_O_2_	−1.081	2.244 (Ag-O)	0.151
Au-Ti_3_C_2_O_2_	−1.460	1.565 (Au-H)	0.122
Ti_3_C_2_F_2_	−0.347	2.700 (C-F)	−0.027
Ag-Ti_3_C_2_F_2_	−0.489	2.214 (Ag-O)	0.048
Au-Ti_3_C_2_F_2_	−0.339	2.115 (Au-H)	0.020
Ti_3_C_2_(OH)_2_	−3.665	1.029 (O-H)	−0.223
Ag-Ti_3_C_2_(OH)_2_	−0.303	2.991 (Ag-H)	−0.183
Au-Ti_3_C_2_(OH)_2_	−0.951	2.898 (Au-H)	−0.117

**Table 4:** Comparative parameters of C_6_H_6_ adsorption on the intrinsic and modified Ti_3_C_2_T_x_ surfaces.

Structure	*E*_ad_ (eV)	Distance (Å)	*Q_t_* (*e*)
Ti_3_C_2_O_2_	−1.298	3.124 (O-C)	0.0752
Ag-Ti_3_C_2_O_2_	−1.852	2.347 (Ag-C)	0.167
Au-Ti_3_C_2_O_2_	−2.467	2.217 (Au-C)	0.197
Ti_3_C_2_F_2_	−1.108	3.057 (F-C)	−0.030
Ag-Ti_3_C_2_F_2_	−1.297	2.330 (Ag-C)	0.140
Au-Ti_3_C_2_F_2_	−1.211	2.241 (Au-C)	0.099
Ti_3_C_2_(OH)_2_	−1.444	2.196 (H-H)	−0.180
Ag-Ti_3_C_2_(OH)_2_	−1.395	2.763 (Ag-H) 2.176 (H-C)	−0.059
Au-Ti_3_C_2_(OH)_2_	−1.431	2.697 (Au-H) 2.301 (H-H)	−0.040

**Table 5:** Comparative parameters of the HCHO and C_6_H_6_ adsorptions on different materials.

Gas	Material	*E*_ad_ (eV)	Distance (Å)	Ref.
HCHO	Al-MnO_2_	−2.282	1.722	[38]
	Fe-MoS_2_	−1.720	/	[39]
	Al-C_2_N	−2.754	2.008	[40]
	Au-Ti_3_C_2_O_2_	−1.460	1.565	This work
	Ag-Ti_3_C_2_F_2_	−0.489	2.214	
	Ti_3_C_2_(OH)_2_	−3.665	1.029	
C_6_H_6_	Al-MnO_2_	−2.416	2.156	[38]
	Fe-MoS_2_	−1.870	/	[39]
	Al-C_2_N	−1.595	1.381	[40]
	Au-Ti_3_C_2_O_2_	−2.467	2.217	This work
	Ag-Ti_3_C_2_F_2_	−1.297	2.330	
	Ti_3_C_2_(OH)_2_	−1.444	2.196	

## Data Availability

Data are contained within the article.
